# Cellular Redox Metabolism Is Modulated by the Distinct Localization of Cyclic Nucleotide Phosphodiesterase 5A Isoforms

**DOI:** 10.3390/ijms23158587

**Published:** 2022-08-02

**Authors:** Silvia Cardarelli, Adriana Erica Miele, Federica Campolo, Mara Massimi, Patrizia Mancini, Stefano Biagioni, Fabio Naro, Mauro Giorgi, Michele Saliola

**Affiliations:** 1 Department of Biology and Biotechnology “C. Darwin”, Sapienza University of Rome, Piazzale A. Moro 5, 00185 Rome, Italy; silvia.cardarelli@uniroma1.it (S.C.); stefano.biagioni@uniroma1.it (S.B.); michele.saliola@fondazione.uniroma1.it (M.S.); 2 Department of Biochemical Sciences, Sapienza University of Rome, Piazzale A. Moro 5, 00185 Rome, Italy; 3 UMR 5280 ISA-CNRS-UCBL, Université de Lyon, 5 Rue de La Doua, 69100 Villeurbanne, France; 4 Department of Experimental Medicine, Sapienza University of Rome, Viale Regina Elena 324, 00161 Rome, Italy; federica.campolo@uniroma1.it (F.C.); patrizia.mancini@uniroma1.it (P.M.); 5 Department of Life, Health and Environmental Sciences, University of L’Aquila, Via Vetoio, 67100 L’Aquila, Italy; mara.massimi@univaq.it; 6 Department of Anatomical, Histological, Forensic, and Orthopaedic Sciences, Sapienza University of Rome, Via A. Borelli 50, 00161 Rome, Italy; fabio.naro@uniroma1.it

**Keywords:** cGMP-specific phosphodiesterase, glycolytic/respiratory flux, *Kluyveromyces lactis*, redox balance, rag phenotype

## Abstract

3′-5′ cyclic nucleotide phosphodiesterases (PDEs) are a family of evolutionarily conserved cAMP and/or cGMP hydrolyzing enzymes, components of transduction pathways regulating crucial aspects of cell life. Among them, cGMP-specific PDE5—being a regulator of vascular smooth muscle contraction—is the molecular target of several drugs used to treat erectile dysfunction and pulmonary hypertension. Production of full-length murine PDE5A isoforms in the milk-yeast *Kluyveromyces lactis* showed that the quaternary assembly of MmPDE5A1 is a mixture of dimers and tetramers, while MmPDE5A2 and MmPDE5A3 only assembled as dimers. We showed that the N-terminal peptide is responsible for the tetramer assembly of MmPDE5A1, while that of the MmPDE5A2 is responsible for its mitochondrial localization. Overexpression of the three isoforms alters at different levels the cAMP/cGMP equilibrium as well as the NAD(P)^+^/NAD(P)H balance and induces a metabolic switch from oxidative to fermentative. In particular, the mitochondrial localization of MmPDE5A2 unveiled the existence of a cAMP-cGMP signaling cascade in this organelle, for which we propose a metabolic model that could explain the role of PDE5 in some cardiomyopathies and some of the side effects of its inhibitors.

## 1. Introduction

Cyclic nucleotides (cAMP and cGMP) are components of evolutionarily conserved transduction pathways mediating a large number of biological processes. Temporal and spatial changes in their concentrations convey complex instructions to translate extracellular signals into a large number of downstream biological processes. PDEs, strictly defined as 3′-5′ cyclic nucleotide phosphodiesterases [1], are a superfamily of enzymes that modulate the amplitude and duration of cyclic nucleotide signaling through their hydrolysis to AMP and GMP. PDEs are classified into 11 structurally related families, according to primary structures, affinities for cAMP and cGMP, catalytic properties and sensitivity to regulators and inhibitors [2,3,4]. Moreover, multiple gene promoters, together with alternative splicing, direct the expression of more than 100 isoforms and further contribute to their molecular diversity.

PDEs exhibit a modular structure characterized by a highly conserved C-terminal catalytic domain and a variable N-terminal regulatory domain. The latter contains several structural motifs which confer specificity to each isoform, such as peculiar quaternary structure, subcellular localization, different post-translational modifications, binding sites for allosteric modulators and for interactions with unique scaffolding and regulatory proteins and effectors [2,5,6,7]. The presence of PDE in specific multi-molecular complexes allows the propagation of signals along well-defined pathways, prevents the diffusion of signals, and ensures the specificity of downstream biological responses [8].

PDE5, among cGMP-specific hydrolyzing families, has been localized in vascular smooth muscle, where it participates in the control of vasodilatation [9,10]. PDE5 has been identified as the molecular target of several well-known drugs used to treat human diseases such as erectile dysfunction and pulmonary hypertension [11]. Lately, its action has been extended to learning/memory processes, heart failure, cardio-vascular diseases, human breast and thyroid cancers, and many other pathologies [11,12,13,14,15,16,17,18].

In mammals, three isoforms of PDE5 have been identified—called A1, A2 and A3—which show similar cGMP catalytic activities, while differing in the very first N-terminal amino acids [19]. In *Mus musculus* PDE5A1 has the longest N-terminal extension (41 aa), MmPDE5A2 has 10 aa before the common part and MmPDE5A3 has no extension (Table 1). Although PDE5A1 and PDE5A2 are widely expressed, whereas PDE5A3 seems restricted to very few specific tissues [20], the specific role of the three almost identical isoforms has up to now not been clarified.

Recently, the entire murine PDE5A isozyme family was characterized [20] and produced in large amounts in the anaerobic facultative *Kluyveromyces lactis* yeast, allowing a thorough characterization of the purified proteins in vitro [21]. Moreover, we identified the presence of tetramers in the assembly of MmPDE5A1, in addition to homodimers—both in yeast and in rat platelets—suggesting a further modulating control of this enzyme as a component of large signaling complexes [22].

Finally, in order to evaluate the role of these isoforms in cell signaling and homeostasis [21,22,23,24], these activities were studied in the yeast *Saccharomyces cerevisiae* [23] and in vivo in murine cardiomyocyte cells [20].

Indeed, in our previous studies, we found that single copy expression of the *MmPde5a1* gene in *S. cerevisiae* altered the endogenous cAMP/cGMP equilibrium and affected the fermentative–respiratory balance, thus implicitly confirming its modulating role in yeast metabolism [23].

Since PDE5 activity has a particular relevance in human physiology being involved in many pathological processes and human diseases, in this paper—using *K. lactis* as a model organism—we evidenced for the first time a specific role of each isoform in the control, modulation, and maintenance of cellular metabolism.

In particular, we present evidence that: (a) the extra N-terminal peptide is able to modify the quaternary structure and the intracellular localization of PDE5A isoforms; (b) redox ligands affect protein flexibility; (c) the localization of the isoforms affects the cytosolic NAD(P)^+^/NAD(P)H redox balance, altering the glycolytic/aerobic metabolic flux; (d) mitochondria, central components of cellular metabolism, are indeed regulated by cGMP as much as cAMP via PKA signaling.

## 2. Results

### 2.1. Quaternary Structure of Recombinant MmPDE5A Isoforms

The three isoforms of MmPDE5A have been individually expressed in *K. lactis* and purified as previously reported [21,22]. All three isoforms are active as cGMP hydrolyzing enzymes. Their catalytic constants are practically the same (K_M_ ranging from 1.08 to 1.1 μM; specific activity V_max_ = 1.8 μmol/min/mg) and comparable with data reported in the literature [1,2,11,19,21,22].

**Figure 1:**
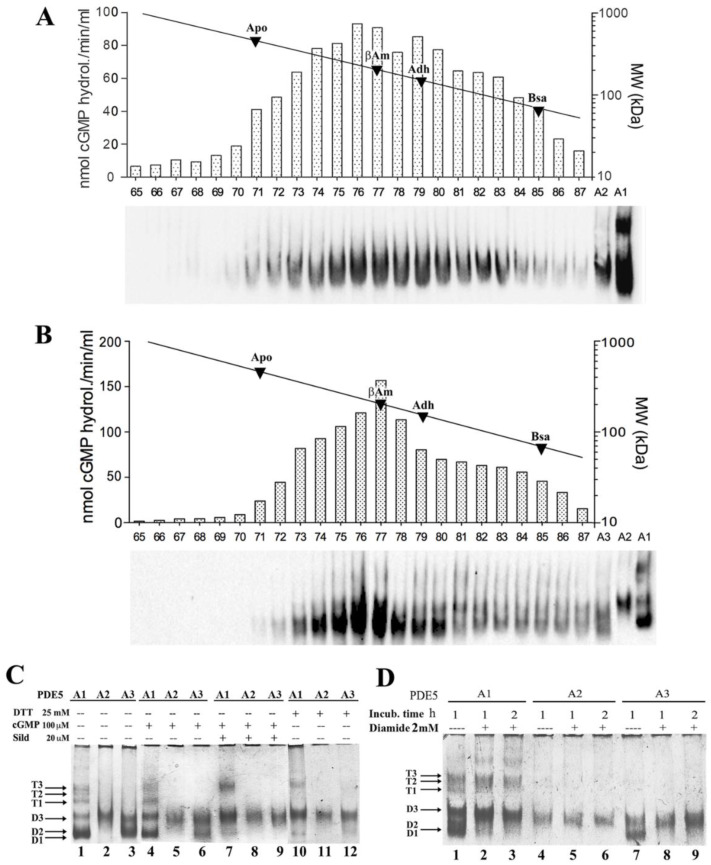
cGMP activity and quaternary assembly of purified recombinant MmPDE5 isoforms. A calibration curve with known MW markers has been superimposed on panels (**A**,**B**): apoferritin (Apo, 443 kDa), β-amylase (βAm, 200 kDa), alcohol dehydrogenase (Adh, 150 kDa), bovine serum albumin (BSA, 66 kDa). (**A**) cGMP-hydrolysing activity and native WB from size exclusion chromatography (SEC) eluted fractions of purified MmPDE5A2. (**B**) cGMP-hydrolyzing activity and native WB from SEC-eluted fractions of purified MmPDE5A3. Fractions were obtained from 300 μg of affinity purified MmPDE5A2 and MmPDE5A3 applied on a Superose 12 HR 10/30 column and eluted at a flow rate of 0.20 mL/min. This SEC fractionation has been repeated at each purification and one representative example is shown in these figures. (**C**,**D**) Native PAGE pattern of purified MmPDE5A1, A2 and A3 stained with Coomassie (~1.0 μg). The proteins were pre-incubated for up to 3 h at 30 °C with dithiotreitol (DTT), cGMP, Sildenafil (Sild) (**C**), and diamide (**D**) at the concentration specified in the figures. D1, D2, and D3 indicate the three conformational forms of the dimer and T1, T2, and T3 those of the tetramer. Native page analyses of MmPDE5 isoforms under different conditions/incubations with different reagents have been performed and repeated at least five times. One representative example is shown for both figures (**C**,**D**).

Unlike MmPDE5A1, which assembles as both dimers and tetramers [22], we showed that MmPDE5A2 and MmPDE5A3 only assemble as dimers, independently of the presence of effectors, ligands, and inhibitors (Figure 1).

However, the structural flexibility of the dimers is different between MmPDE5A2 and MmPDE5A3, as can be seen in the size exclusion chromatography (SEC) elution profiles and native polyacrylamide gel electrophoresis (PAGE) (Figure 1). In fact, MmPDE5A2 elutes as a larger gaussian (fractions 71–85 in Figure 1A), while the elution peak of MmPDE5A3 is narrower (fractions 73–80, Figure 1B). Nevertheless, in both cases the peak is centered at around 200 kDa, as they only differ in the first 10 amino acids, MmPDE5A3 being devoid of the extra N-terminal peptide (Table 1).

Moreover, the migration mobility of these isoforms in native PAGE showed that MmPDE5A2 is a compact dimer unaffected by ligand, inhibitor, reducing, or oxidizing agents. MmPDE5A3 dimer is as flexible as MmPDE5A1 dimer and can be as well rigidified by the addition of sildenafil, dithiotreitol, and diamide (Figure 1C,D).

In these experiments, MmPDE5A1 migrates, as previously reported, both as multiple tetrameric and dimeric conformations, respectively named T1–T3 and D1–D3, according to previous data [22,25] (Figure 1C, lane 1). Consistent with SEC data, MmPDE5A2 and MmPDE5A3 showed only the faster migrating bands, as compared to MmPDE5A1 (Figure 1C, lanes 2–3). Interestingly, MmPDE5A2 displayed a unique band with migration properties resembling those of the D3 band of both MmPDE5A1 and MmPDE5A3 (Figure 1C, lanes 1–3).

### 2.2. Overexpression of MmPde5a2 Induces a Mutation That Affects Glucose Oxidation in K. lactis

**Figure 2:**
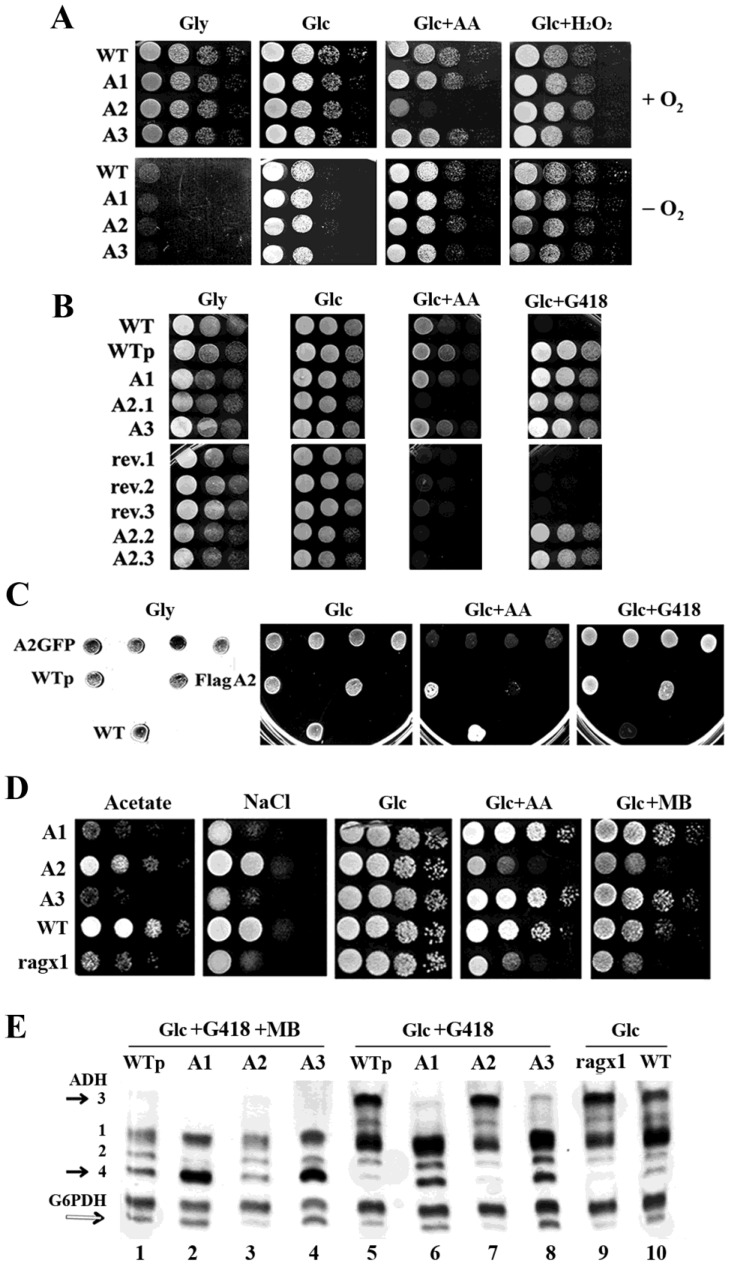
Growth tests of *K. lactis* wild type strain (CBS2359) and its transformants with MmPDE5A1, MmPDE5A2, MmPDE5A3, and in-gel native staining assays of their protein extracts. (**A**) Cells were incubated in the presence of oxygen (+O_2_) or under anaerobic conditions (−O_2_). (**B**) Growth test of the above strains plus selected colonies of the PDE5A2-strain in which the p3xFlagPde5A2 has been lost (revertant rev.1, rev.2, rev.3). A2.2 and A2.3 are two other re-transformants of the WT (CBS2359) strain with *p3xFlagPde5A2*. (**C**) Growth test of WT, WT + empty vector (WTp), *pPde5A2gfp*, and *p3xFlagPde5A2* transformed strains. All strains were grown in YP + 2% glucose (Glc), plus 5 mM H_2_O_2_, (Glc + H_2_O_2_) plus 100 μM G418 (Glc + G418), or 5% glucose plus 5 μM antimycin A (Glc + AA), 2% glycerol (Gly). (**D**) Growth test of ragx1, WT and transformed strains with MmPDE5A1 (A1), MmPDE5A2 (A2), MmPDE5A3 (A3). Strains were grown in YP medium + 4 mM Acetate (Acetate), glucose 2% (Glc) containing 1.2 M NaCl (NaCl) or 30 μM methylene blue (Glc + MB) or 5% glucose plus 5 μM antimycin A (Glc + AA). Cells, grown to early stationary phase, were adjusted to 10^8^ cells mL^−1^ and 5 μL of serial 10-fold dilutions were spotted onto the indicated medium. Growth was followed for 3–5 days. The initial concentration was 10^7^ cells mL^−1^. (**E**) *K. lactis* in-gel native staining for alcohol dehydrogenase (ADH) and Glc-6-phosphate dehydrogenase (G6PDH). Cells were grown to early stationary phase in YP containing 2% glucose + MB + G418 (Glc + MB + G418), 2%glucose + G418 (Glc + G418) or 2% glucose (Glc). Extracts from these cultures, fractioned in native PAGE, were stained for ADH and G6PDH. Black arrows indicate the migrating positions of KlAdh3 and KlAdh4, white arrow indicates the faster migrating band of G6PDH that corresponds to the less active dimeric form. Native PAGE analyses of ADH and G6PDH activities from *K. lactis* cells harboring PDE5 isoforms have been repeated at least three times. S.D. among the same bands of each lane was comprised between 4% and 13%. One representative native gel is shown in the figure.

To investigate whether the different migrating properties of MmPDE5A2 observed in Figure 1C, as compared to those of MmPDE5A1 and A3, were possibly due to its heterologous expression, we performed a phenotypical analysis of *K. lactis* strains individually expressing each isoform (Figure 2). To this end, we performed serial dilution growth tests in the presence of respiratory and fermentative carbon sources, mitochondrial respiratory chain (mRC) inhibitors, antibiotics, and oxidative and reducing stress conditions. As can be seen in Figure 2A, only the strain transformed with the *MmPde5a2* plasmid had a drastically reduced growth on antimycin A (AA), a known inhibitor of Complex III of the mRC. Normal growth could be resumed under hypoxic conditions, suggesting the activation of alternative fermentative pathways to bypass the O_2_-dependent regulation of the mRC (Figure 2A).

In fact, *K. lactis* is an anaerobic facultative Crabtree-negative yeast [26], in which fermentation only occurs when oxygen becomes limiting [27]. Moreover, it has been reported that *K. lactis* strains harboring mutations along the glycolytic/fermentative pathways are unable to grow on glucose (Glc), whenever the mRC is blocked by inhibitors. This complex phenotype, called Rag^−^ (where Rag^+^ = Resistant to antibiotics on glucose) [28], comprises a growing number of complementation groups (more than 20 genes), which includes Glc transporter and sensors, glycolytic genes, transcription factors, and many other genes regulating Glc metabolism [29,30].

In order to test whether there was a direct link between the sensitivity to AA of the host and the overexpression of MmPDE5A2, a few colonies—following the loss of the plasmid—were selected by their inability to grow on G418 (rev.1, rev.2, rev.3 in Figure 2B).

In parallel, we also re-transformed fresh wild type *K. lactis* cells with each of the *MmPde5* plasmids (A1, A2, A3) and with an empty plasmid (WTp). As can be seen in Figure 2B, only the strains that have lost the *MmPde5a2* plasmid and the ones re-transformed with the same construct were still unable to grow on antimycin A, thus displaying a permanent Rag^−^ phenotype, i.e., a *rag* mutation fixed in the host genome. The result was unchanged if the host was transformed with a plasmid expressing PDE5A2-GFP fusion protein, in which MmPDE5A2 is tagged at the C-terminus instead of the N-terminus (*FlagPde5a2*). Therefore, overexpression of MmPDE5A2 in *K. lactis* induces mutations that we called *ragx1* (Figure 2C,D), which is independent of the nature and position of the tag.

### 2.3. Overexpression of PDE5A Isoforms Affects the Cytosolic NAD^+^/NADH–NADP^+^/NADPH Redox Balance

Rag^−^ strains, in addition to antimycin A, are also sensitive to osmotic stress (NaCl) [31] and to methylene blue (MB), a reagent known to interfere with cytosolic NADPH oxidation in *K. lactis*. MB was the first synthetic drug, a reagent that under physiological conditions is a blue cation, which undergoes a catalytic redox cycle: MB is reduced by NADPH to give an uncharged colorless compound (for a review see [32]). MB has been used in many different medical applications and as an inhibitor of NADPH-activated nitric oxide-stimulated soluble guanylyl cyclase, widely used for the control of cGMP-mediated processes [33].

To better characterize the growth of the *ragx1* mutant, cells were grown in serial dilution tests under osmotic stress, in the presence of MB, antimycin A, and acetate growth conditions (Figure 2D).

Unexpectedly, differently from the growth observed on antimycin A and MB (Figure 2D), which were similar to the results shown in Figure 2A, the growth of PDE5A1, A2, and A3 on acetate and under osmotic stress conditions was totally different between the parental WT of the strains expressing A1 and A3 and ragx1 that is the parental of the strain expressing A2. These results indicated that both PDE5A1 and PDE5A3, being unable to accumulate glycerol to counteract the osmotic stress, increased the fermentative capabilities of the WT CBS2359 strain. Conversely, PDE5A2 affected the metabolism of its parental *ragx1* mutant and, at the same time, took the control of the signaling to resist osmotic stress and grow on acetate. These results imply that PDE5 isoforms are able to control yeast metabolism through the cAMP-cGMP/protein kinase A (PKA) pathways [23,34].

In order to check the oxidizing vs. fermentative metabolism of WT and ragx1 strains, we used KlAdh3 and KlAdh4—two endogenous alcohol dehydrogenase (ADH) activities—as markers of respiratory and fermentative metabolism, respectively [35].

**Figure 3:**
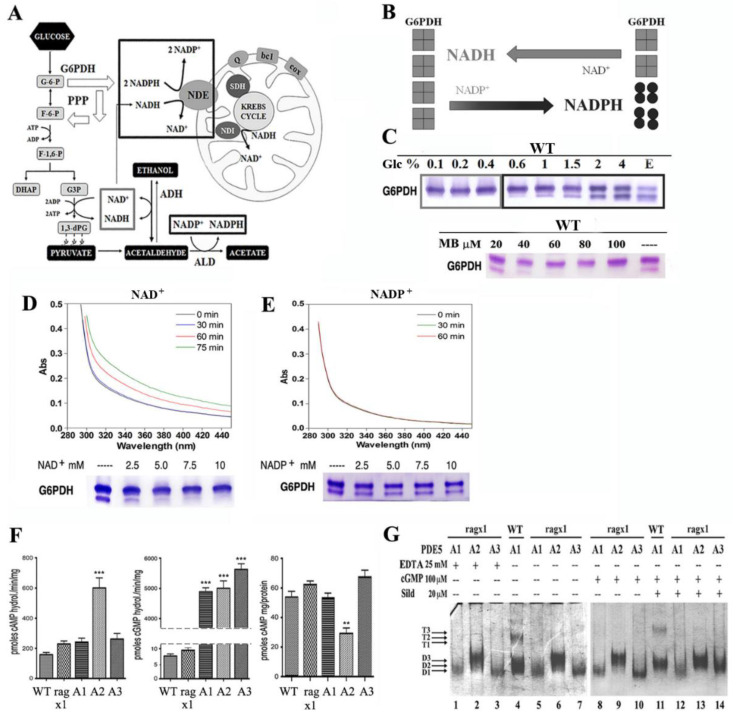
Role of the coenzymes NAD(P)^+^/NAD(P)H in the onset of fermentation in the *K. lactis* WT strain, quantification of cyclic nucleotide PDE activity, cAMP content and effects of the *ragx1* mutation on the quaternary structure of MmPDE5 isoforms. (**A**) Schematic summary of the role of NAD^+^ vs. NADP^+^ during the glycolytic–fermentative metabolism, their implication in the pentose phosphate pathway (PPP), their oxidation by NDE1 (which has higher affinity for NADH than for NADPH). (**B**) A model showing the opposite role of the coenzymes on the assembly and activity of G6PDH (**C**) Effect of cytosolic NAD(P)H on G6DPH isoforms during the respiratory to fermentative transition (from Glc 0.1% to 4%; E stands for Ethanol). Effect of NADPH oxidation by MB during fermentation (YP^+^ 2% Glc) on G6PDH isoforms. (**D**) Absorbance spectra and G6PDH content of WT cell extracts supplemented with increasing concentrations of NAD^+^ (up to 10 mM). (**E**) Absorbance spectra and G6PDH content of WT cell extracts supplemented with increasing concentrations of NADP^+^ (up to 10 mM). (**F**) Extracts from 10 mL cultures of ragx1, WT, MmPDE5A1 (A1), MmPDE5A2 (A2), and MmPDE5A3 (A3) transformed strains grown to early stationary phase were used to determine the cAMP hydrolyzing activity (left panel), cGMP hydrolyzing activity (center panel) and cAMP content (right panel). Reported values are the means from three independent determinations (S.D. was always between 2% and 12%). Student’s *t*-test was used to determined statistical significance with respect to WT: ** *p* < 0.01; *** *p* < 0.001 (**G**) Quaternary assembly of recombinant MmPDE5A isomers produced in the *ragx1* mutant. Native PAGE pattern of purified MmPDE5A1, MmPDE5A2, and MmPDE5A3 stained with Coomassie (~1.0 μg). Legend as in Figure 1C,D. MmPDE5A1 protein purified from the WT strain has been loaded as a control. Native page analyses of MmPDE5 isoforms under different conditions/incubations are the means of at least three independent experiments. One representative gel is shown in the figure.

As shown in the native PAGE (ADH pattern analysis of Figure 2E), strains expressing MmPDE5A1 and A3, grown in the presence of MB, showed higher levels of Kladh4 and increased fermentative capabilities (Figure 2E, lanes 2 and 4), as compared to WTp and MmPDE5A2 (Figure 2E, lanes 1 and 3). KlAdh4 was also very abundant in A1 and A3 under selective conditions (Glc + G418) (Figure 2E, lanes 6 and 8), but almost undetectable in WTp and A2 (lanes 5 and 7). The overexpression of the three isoforms in *K. lactis* led us to two main conclusions: (a) in *K. lactis* O_2_ directly controls the mRC (Figure 2A) [27], since MB interferes with the cytosolic reoxidation of NADPH, increased fermentation is shown by the repression of the *KlADH3* gene (Figure 2E, lanes 1–4). Indeed, in Glc conditions without MB, the amounts of KlAdh3 reflected the levels of the respiratory and fermentative metabolism of a culture (Figure 2E, lanes 5–10) and its transition from one condition to the other (as shown in [35]). (b) The amount of KlAdh4 (black arrow in Figure 2E) is linked to the amount of the faster migrating band of Glc-6-phosphate dehydrogenase (G6PDH) (white arrow in Figure 2E), the first enzyme of the pentose phosphate pathway (PPP), which underwent a tetramer to dimer switch, as shown by the partial conversion of the slow migrating G6PDH band into the faster one (Figure 2E, lanes 2, 4, 6, 8 versus lanes 3, 7, 9 and Figure 3). It had previously been reported that the G6PDH tetramer is the active form, while the dimer is inactive [36], schematized in Figure 3B.

Diversion of the glycolytic flux to the PPP under impaired fermentation is a rescue mechanism used by *K. lactis* to avoid the accumulation of cytosolic NADPH excess, a condition necessary during fermentation to reduce competition for the oxidation of cytosolic NADH [36,37].

To better understand the metabolic changes induced in the PDE5A1, A2, and A3 transformants as compared with their parental WT and ragx1 strains, we investigated the role of redox coenzymes NAD^+^/NADH and NADP^+^/NADPH in the onset of fermentation, by growing the WT (CBS2359) on rich medium (YP) containing 0.1% up to 4% Glc (Figure 3A–E).

The scheme in Figure 3A shows the metabolic activities involved in the cytosolic cofactor balance during fermentation and their competitive oxidation by KlNde1, according to [38,39,40]. KlNde1 is a NAD(P)H trans-dehydrogenase located on the inner mitochondrial membrane (with its active site towards the intermembrane space), able to oxidize both cofactors, although NADH has a higher affinity as compared to NADPH [41]. KlNde1, together with the trans-dehydrogenase KlNdi1—specific for NADH and whose active site is in the matrix—substitutes the Complex I of the mRC in many yeasts.

Differently from *S. cerevisiae*, *K. lactis* has no catabolite repression and is able to use at the same time both glucose and accumulated ethanol, which leads to competition of NADH and NADPH for their re-oxidation by KlNde1. As fermentation increases, we observed the partial dimerization of G6PDH, limiting the amount of NADPH to be oxidized as compared to NADH (Figure 3B).

These data are further confirmed by the growth on ethanol (Figure 3C, last lane) which produces an equal cytosolic amount of both cofactors. Therefore, since MB selectively reduces the amounts of NADPH re-oxidized by KlNde1, it acts as an inhibitor of the mRC, at least in *K. lactis*. In fact, extracts grown in YP containing 2%Glc with MB excess (MB ≥ 30 μM)—beside the block of the mRC—show oxidative stress, which reverts G6PDH to the active tetramer (Figure 3C).

The same effect (G6PDH tetramerization) is achieved in vitro by incubating the cell extracts overnight with NAD^+^ (up to 10 mM) (Figure 3D). In contrast, the incubation with NADP^+^ was unable to modify the G6PDH pattern (Figure 3E). In parallel, the analysis of the absorbance spectra of the same extracts only responded to the addition of 0.3 mM NAD^+^ (Figure 3D), but not to that of NADP^+^ (Figure 3E). In conclusion, the dimer/tetramer assembly of G6PDH is a good marker to follow the metabolic glycolytic flux in *K. lactis* and, therefore, the metabolic alterations produced by MmPDE5 isoforms overexpression.

### 2.4. Over-Expression of PDE5A2 Affects Both cAMP Content and cAMP-Hydrolyzing Activity

We also measured the cAMP content in all strains, as well as the hydrolyzing activities specific to cAMP and cGMP. Indeed, the *ragx1* mutant overexpressing MmPDE5A2 had three times more cAMP-specific activity (Figure 3F, left), and two times less cAMP content than WT and MmPDE5A1 and MmPDE5A3 overexpressing strains (Figure 3F, right). Moreover, the parental ragx1 strain had cAMP levels similar to the WT, A1, and A3 strains (Figure 3F, left). This result demonstrates a direct and specific effect of MmPDE5A2 on cAMP levels.

In contrast, the amounts of cGMP hydrolyzing activity are more than 500 times higher in the strains over-expressing the three isoforms, as compared to WT and ragx1 control strains (Figure 3F, central histogram).

### 2.5. The ragx1 Mutant Context Modifies the Quaternary Structure of MmPDE5A Isoforms

Since we had noticed that recombinant MmPDE5A2 freshly purified was an active rigid dimer insensitive to ligands and redox chemicals, even at very high concentrations (Figure 1C,D), we tested whether the ragx1 strain was able to modify also the structure of MmPDE5A1 and MmPDE5A3, once overexpressed. To our surprise, both proteins purified from the ragx1 strain were only dimeric and unresponsive to ligands (Figure 3G). Moreover, the migrating properties were also altered, MmPDE5A1 and A3 only migrated as the D1 band, while MmPDE5A2 migrated as the D3 band.

Altogether, these results indicate that overexpression of MmPDE5A2 gives rise to the *ragx1* mutation by inducing a permanent imbalance in the cyclic nucleotides equilibrium and in the glycolytic–fermentative metabolism of the host. Conversely, *ragx1* affects the quaternary structure of the three isoforms, possibly by interfering with post-translational modifications or accessory regulatory proteins.

### 2.6. MmPDE5A2 Is Localized in the Mitochondria

The specific role of MmPDE5A2 in the appearance of *ragx1* suggested a different subcellular localization as compared to the other two isoforms. By using GFP-tagged isoform constructs and fluorescence microscopy, we performed in vivo localization studies as previously reported in [19].

**Figure 4:**
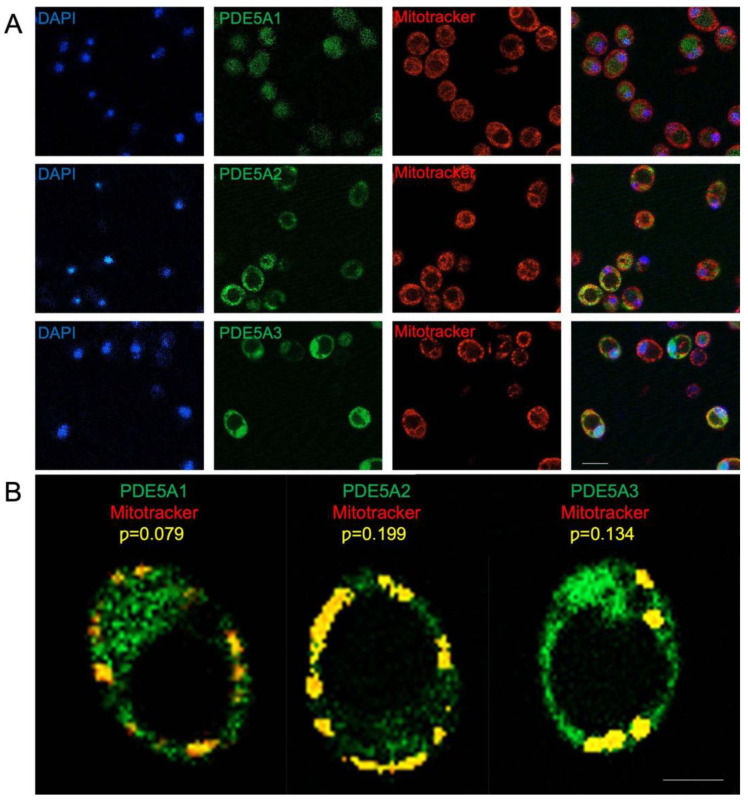
Fluorescence microscopy images of *K. lactis* cells overexpressing MmPDE5AGFP isoforms. (**A**) Intracellular localization of MmPDE5A1, A2, and A3 isoforms revealed in cell transformed with GFP-tagged isoform constructs (green). Cultures were grown to early stationary phase in YP containing 2%Glc + G418; cells stained with DAPI (blue) and MitoTracker (red) were visualized under the confocal microscope. Scale bar: 10 µm. (**B**) Colocalization analysis on *K. lactis* cells overexpressing MmPDE5A-GFP isoforms. Cells overexpressing GFP-tagged MmPDE5A1, A2, and A3 isoforms (green) were grown to early stationary phase in 2%Glc + G418 and stained with MitoTracker (red). Images showing a representative single cell, were acquired using a Zeiss LSM 800 System. Colocalization signal (yellow) was quantified using the Pearson correlation coefficient (*p*). Original magnification 20×; Inset 10×; Scale bar: 5 μm.

Microscopy analysis of the GFP signal (Figure 4A) shows that MmPDE5A1 displays a mainly cytosolic localization, while MmPDE5A3 has a more diffuse signal, suggesting its broader localization between cytosol and nucleus, thus indicating their highly reduced respiratory capabilities and massive switch towards fermentation.

In contrast, the GFP signal of MmPDE5A2 is restrained in circular dots, suggesting a specific localization into the mitochondria. To confirm this hypothesis, we analyzed the mitotracker signal in these cells. After green-red signals superposition (Figure 4A), we concluded that MmPDE5A2 is mainly localized into the mitochondria. To quantify this signal, we performed a colocalization analysis between mitotracker and GFP (Figure 4B). Cells from three independent experiments were used to determine Pearson’s correlation coeffient (*p*) as following: PDE5A1 *p* = 0.079 (±0.004); PDE5A2 *p* = 0.199 (±0.014); PDE5A3 *p* = 0.134 (±0.007). Statistical significance was calculated using the ANOVA one-way test with Holm–Sidak’s post hoc comparison. ** *p* = 0.007 (PDE5A2 vs. PDE5A1).

However, further investigation will be required to establish in which mithochondrial subcellular compartment(s) MmPDE5A2 is present (i.e., the intermembrane space or the matrix).

Although we cannot exclude additional localizations of the three isoforms, the 10 extra N-terminal amino acids present in MmPDE5A2 seem to be necessary and sufficient *per se* to target this cGMP hydrolyzing isoform to the mitochondria and the respiratory machinery. Our results are consistent with the mitochondrial localization of the rat PDE2A2 isoform [42].

Finally, a computational analysis was undertaken on their N-terminal sequences for the presence of mitochondrial targeting sequences (MTS) using MitoProtII [43] to confirm the different localization of the three MmPDE5 isoforms. This analysis showed that MmPDE5A2 N-terminus contains a putative MTS (28.61% probability), while MmPDE5A1 and mmPDE5A3 displayed a much lower probability to be imported into mitochondria (8.36% and 8.59% probability, respectively).

## 3. Discussion

### 3.1. Quaternary Assembly and Cellular Localization of MmPDE5 Isoforms

Using *K. lactis* as a model organism, the full-length isoforms of murine phosphodiesterase 5 (MmPDE5) have been successfully purified and studied, evidencing for the first time a specific role of each isoform in the control, modulation, and maintenance of cellular metabolism. The three isoforms differ only in the very extra N-terminal sequence, which is composed of 41 amino acids in A1, 10 in A2 and none in A3 (Table 1). MmPDE5A1 and MmPDE5A3 are translations of the same mRNA, starting at two different AUG codons, while MmPDE5A2 is an alternatively spliced isoform [20]. Indeed, this extra peptide is able to drive the distinct quaternary assembly of the three isoforms, namely homo-dimer and homo-tetramer in MmPDE5A1 (as already reported in [22]), and only homodimer in MmPDE5A2 and MmPDE5A3, as shown by SEC, native WB, and PAGE (Figure 1). Therefore, the common sequence is responsible for the dimerization, while the longest N-terminal extension drives the tetramerization.

Another structural characteristic we have highlighted is that MmPDE5A1 and MmPDE5A3 remain quite flexible and explore a larger conformational space, both in dimers and tetramers (when present), as shown by native PAGE (Figure 1C,D and Figure 3G). These conformations are sensitive to effectors, inhibitors, reducing and oxidizing agents, which have the effect of rigidifying each quaternary structure. In contrast, MmPDE5A2 is a rigid dimer (Figure 1C,D and Figure 3G), totally insensitive to ligands (cGMP, sildenafil) and redox agents (DTT, diamide).

Finally, we evidenced a different localization of the three isoforms, mainly cytosolic for MmPDE5A1, while MmPDE5A3 has a more diffuse signal, suggesting its broader localization between cytosol and nucleus. In contrast, MmPDE5A2 seems to be not only localized in mitochondrial compartments (Figure 4) but is mutagenic, inducing in the host a mutation (*ragx1*) due to a permanent imbalance in the cyclic nucleotides equilibrium and, as a consequence, in the glycolytic–fermentative metabolism (Figure 3F). Moreover, the *ragx1* context rigidifies the quaternary structure of the three MmPDE5A isoforms, which become unable to control and modulate *K. lactis* cell metabolism (Figure 3G).

Although further experiments will be necessary to access the localization of PDE5 isoforms in higher eukaryotic cells, these results might explain why the N-terminal signal peptide is evolutionary conserved among mammalian species [20]. Clearly, the *K. lactis* model constitutes a limitation in the study of human PDE5, but these metabolic effects on yeast—if confirmed in mammalian cells—might suggest clinical strategies for severe pathologies determined by their altered expression or balance in human tissue and organs.

### 3.2. Metabolic Role of MmPDE5A2 in K. lactis

**Figure 5:**
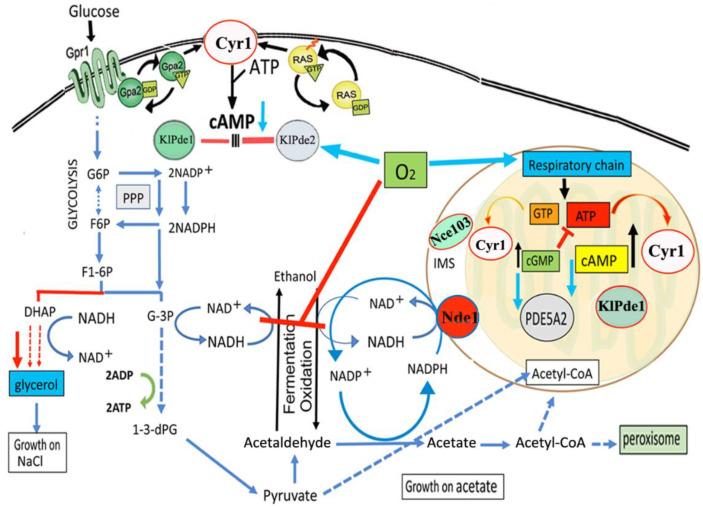
Schematic model of the metabolic changes induced in *K. lactis* by MmPDE5A2. The mitochondrial localization of MmPDE5A2 highlighted the existence of a Cyr1-cAMP/cGMP signaling system in mitochondria dedicated to balancing O_2_ availability and oxidative phosphorylation with Krebs cycle turnover. cGMP hydrolysis by MmPDE5A2 activates an O_2_-dependent mechanism, increasing the production of ATP by the respiratory chain while at the same time blocking the fermentation, either directly or through the cAMP-specific endogenous KlPde2. Under physiological conditions, the mitochondrial role of PDE5A2 may be fulfilled by the endogenous KlPde1. The adenylate cyclase Cyr1 is controlled by Ras/Gpa2-Gpr1 and NAD(P)H is re-oxidized by the trans-dehydrogenase Nde1. The carbonic anhydrase Nce103 is located also in the inner mitochondrial space (IMS). G6P, glucose-6 phosphate; F6P, fructose-6 phosphate, F1-6P, fructose1-6 diphosphate; G-3P, glyceraldehyde-3 phosphate; 1-3dPG, 1-3diphosphoglycerate; DHAP, dihydroxyacetone phosphate.

According to our results and in line with what is reported in mitochondria of rat liver and brain, we hypothesize the presence of a cGMP signaling in *K. lactis* mitochondria (Figure 5).

In rat mitochondria, a complete cAMP signaling activated by bicarbonate/CO_2_ stimulates the mRC, hence linking the oxidative phosphorylation to the Krebs cycle [42]. In this cascade, CO_2_ from the Krebs cycle—converted into bicarbonate by the carbonic anhydrase—stimulates a soluble adenylate cyclase (sAC) to produce cAMP, which in turn stimulates the mRC. The signal is turned off by cAMP hydrolysis catalyzed by phosphodiesterase 2 (PDE2A2).

The effect of PDE2A2 described in rat is similar to the effect of MmPDE5A2 in yeast mitochondria. MmPDE5A2 induces in *K. lactis* the *ragx1* mutation, which is responsible at the same time for the O_2_-dependent block of fermentation and for the activation of the respiratory chain. The metabolism becomes mixed oxidative-fermentative (as already reported by [44]), ATP is produced by the mitochondrial oxidation of cytosolic NAD(P)H by Nde1, while the NADH redox excess leads to accumulation of glycerol, as suggested by the growth on NaCl (see Figure 2D).

Therefore, we might conclude that in the MmPDE5A2 transformed strain the cAMP-PKA pathway is either partially inhibited or blocked, due to an imbalance of cyclic nucleotides, while in the *ragx1* mutant, after the loss of the plasmid, this equilibrium is somehow re-established.

In metabolic terms, overexpression of MmPDE5A1 and MmPDE5A3 increases the fermentative capabilities and reduces the respiratory metabolism of the host. Overexpression of MmPDE5A2 provokes a massive disequilibrium of cyclic nucleotides and cofactors, which in turn induces the *ragx1* mutation. The mutations that give rise to the Rag^−^ phenotype are indeed complex; however, here we show for the first time that *ragx1* imbalance of NAD(P)^+^/NAD(P)H, common to many *rag* mutants [28,44], is directly linked to the cAMP and cGMP imbalance.

Since the MmPDE5 isoforms transformed into the *ragx1* mutant are rigidified, we hypothesize a link between the determinants of the quaternary flexibility of MmPDE5 isoforms and cyclic nucleotides (dis)equilibrium, which in turn is linked to cytosolic NAD^+^/NADH − NADP^+^/NADPH redox balance and hence to the energy state of the cell.

In this model (Figure 5), the balance between cGMP and cAMP is required to couple O_2_ availability and oxidative phosphorylation to Krebs cycle turnover. This balance is important in anaerobic facultative yeasts, where Cyr1 (a guanylyl and adenylyl cyclase) is involved in the synthesis of both cAMP and cGMP in many cellular compartments, including mitochondria [45]. Based on our results, we suggest that—under physiological conditions—the endogenous *K. lactis* cGMP/cAMP-hydrolyzing enzyme KlPde1 controls both nucleotides in yeast mitochondria [46], which might indicate the evolutionary conserved existence of this pathway in all eukaryotes.

The endogenous *K. lactis* cAMP-specific KlPde2 is controlled and activated by O_2_ (Figure 5) and responsible for the reduced content of cAMP (Figure 3F right panel). Nce103, a carbonic anhydrase also localized in mitochondria, might be involved in the activation of Cyr1 in analogy with sAC in rat mitochondria. Moreover, the activation of acetate, the direct precursor of Acetyl-CoA, is directed through ACS (Acetyl-CoA synthase) genes by PDE5A2. Therefore, overexpression of MmPDE5A2 highlighted the presence of a signaling cascade in yeast mitochondria functionally analogous to rat mitochondria. Thus, it confirms the central role of mitochondria as regulators of cellular metabolism.

Conversely, the increased fermentation observed in MmPDE5A1- and A3- overexpressing strains (Figure 2E) confirmed the existence of a cytosolic cAMP/cGMP signaling cascade, activated by the hydrolysis of cGMP, as we previously described in *S. cerevisiae* [23].

## 4. Materials and Methods

### 4.1. Strains, Media, Culture Conditions, and Vectors

The genotype of the *K. lactis* CBS2359 strain (https://wi.knaw.nl (accessed on 1 June 2022)), media, growth conditions, and all the other materials and protocols used for the heterologous production, affinity purification, and enzymatic characterization of the recombinant full length MmPDE5A1, MmPDE5A2, and MmPDE5A3 isoforms were previously described [21]. Briefly, *K. lactis* cells harboring the PDE5A constructs under the control of the *KlADH3* promoter were grown under shaking conditions at 28 °C in YP (1% Difco yeast extract, 2% Difco Bacto-peptone) or in minimal medium (6.7 g/L Difco Yeast Nitrogen Base) supplemented by carbon sources at the concentration specified in the text. Geneticin (G418) concentration in selective conditions was 100 mg/mL. Quantitative preparations of the extracts for purification, purity, and biochemical activity/inhibition by sildenafil of PDE5A isoforms have been previously described [21]. Phenotypical analyses of different strains on microtiter plates have been performed by growing *K. lactis* wild type/transformed cells to early stationary phase using growth conditions specified in the text. Cultures were adjusted to 10^8^ cells mL^−1^ and 5 μL of serial 10-fold dilutions were spotted onto the indicated medium. Antimycin A was added in YP medium containing 5% glucose (Glc) at a concentration of 5 μM. Methylene blue was added in YP 2% Glc medium at 30 μM or at the concentration specified in the text. H_2_O_2_ was added at a concentration of 5 μM. Anaerobic conditions on Petri dishes were achieved using the OXOID Anaerobic Gas pack AN0010C (Oxoid Ltd., Basingstoke, Hants, UK), according to the manufacturer’s instructions. The CBS2359/ragx1 strain was isolated from the p3XFlagPDE5A2-CBS2359 transformed strain following loss of the plasmid.

The *K. lactis* pYG137/1 vector, containing the Tn903 transposon required for the selection on G418 in yeast, was used to construct p3XFlagPDE5A1, p3XFlagPDE5A2, and p3XFlagPDE5A3, which contain the *Mus musculus Pde5a1*, *a2*, and *a3* spliced-variants of the *Pde5a* gene fused at their 5′ end to the short DNA sequence coding for the 3XFLAG peptide (Sigma) [21]. pYG137/1 has also been used to construct the pPDE5A1GFP, pPDE5A2GFP, and pPDE5A3GFP vectors containing the three genes in frame with the GFP gene at the C-terminal site [20].

### 4.2. Size Exclusion Chromatography (SEC)

Purified recombinant MmPDE5A1, MmPDE5A2, and MmPDE5A3 prepared from transformed *K. lactis* cells were loaded onto an FPLC Superose 12 HR 10/30 column (GE HealthCare) and eluted at 4 °C at a flow-rate of 0.20 mL/min with 20 mM HEPES buffer, pH 7.0 containing 1 mM EGTA, 5 mM β-mercaptoethanol, 5 mM MgCl_2_, 100 mM NaCl, 2 mM PMSF, and 0.05% v/v Triton X-100. Eluted aliquots of 0.2 mL each were collected and assayed for PDE activity and WB analysis. The molecular weight (MW) of the eluted proteins was estimated following the calibration of the column using thyroglobulin (669 kDa), apoferritin (443 kDa), β-amylase (200 kDa), alcohol dehydrogenase (150 kDa), bovine serum albumin (66 kDa), carbonic anhydrase (29 kDa), and blue dextran (for void volume) as standards.

### 4.3. PDE Enzymatic Assays

PDE activity was measured at 30 °C with the two-step method described in [47] using [^3^H]cGMP or [^3^H]cAMP (Perkin Elmer, Waltham, MA, USA). Aliquots of eluted fraction from SEC were incubated in 60 mM HEPES pH 7.2 assay buffer containing 0.1 mM EGTA, 5 mM MgCl_2_, 0.5 mg/mL bovine serum albumin, and 30 μg/mL soybean trypsin inhibitor, in a final volume of 0.15 mL. The reaction was started by adding tritiated [^3^H]cGMP or [^3^H]cAMP substrate, at a final concentration of 1 μM and stopped by adding 0.1M HCl. The specific activity was quantified at the 10% limit of the total substrate hydrolyzed. Sildenafil was a generous gift from Pfizer (New York, NY, USA).

### 4.4. Native Gel Electrophoresis Analysis

Native polyacrylamide gel electrophoresis (PAGE) was performed with 5% non-denaturing acrylamide gel with a Tris/glycine pH 8.3 running buffer at 4 °C for 60–80 min with a current of 20 mA in a Bio-Rad Mini-Protean electrophoresis apparatus [48]. Each well was loaded with 1.0 μg of purified recombinant MmPDE5A1, MmPDE5A2, or MmPDE5A3, pre-incubated at 30 °C with substrate and/or inhibitor/modifier at the concentrations and time specified in the figures; the activity buffer consisted of 5 μL of 50 mM HEPES pH 7.5, 50 mM NaCl, 15 mM MgCl_2_. Protein bands were visualized by Coomassie-staining. The concentrations of sildenafil and cGMP were higher than therapeutically used in order to demonstrate the conformational structural changes, according to [25].

### 4.5. Native Western Blot Analysis

5 μL of purified MmPDE5A1, MmPDE5A2, and MmPDE5A3 were loaded on a discontinuous native 3% stacking and 5% separating polyacrylamide gel in the absence of denaturing reagents at pH 8.8. After electrophoresis, the proteins were transferred to nitrocellulose membranes (Bio-Rad Laboratories Inc., Hercules, CA, USA). Blots were incubated overnight at 4 °C with rabbit polyclonal anti-PDE5 antibody (1:1000 v/v; Santa Cruz Biotechnology, no. sc-32884). Horseradish peroxidase conjugated anti-rabbit IgG (1:10,000 v/v; Sigma-Aldrich) was used as a secondary antibody (1 h incubation) to reveal the immune-complexes. Bands were visualized using an enhanced chemiluminescence kit (Bio-rad Laboratories Inc., Hercules, CA, USA).

### 4.6. Alcohol Dehydrogenase (ADH) and Glucose 6-Phosphate Dehydrogenase (G6PDH) Native Assays

*K. lactis* cell extracts for ADH and G6PDH staining assays were carried out as previously described [35,48,49]. Staining assays for each specific experiment are the result of at least three independent determinations. Since ADH stained assays reveal the activities of four independent genes (*KlADH1–4*) regulated by different metabolic conditions, their overall patterns constitute a marker of *K. lactis* metabolism [35,48]_._ Moreover, ADH and G6PDH activities were determined in the same gel directly indicating the direction of the glycolytic flux towards fermentation or penthose phosphate/respiration [49].

### 4.7. Determination of cAMP Content

*K. lactis* cells grown at late exponential phase in 10 mL of YP 2%Glc medium were washed in PBS 1× and then broken under shaking conditions with glass beads (⌀ 0.5 mm) in 0.1 M HCl. The lysate was centrifuged at 10,000 g. The collected supernatant was acetylated, according to the instructions of the kit manufacturer (Direct cAMP ELISA kit; Enzo Life Sciences Inc., Farmingdale, NY, USA). Briefly, the kit uses a polyclonal antibody to cyclic nucleotides that binds, in a competitive manner, the cyclic nucleotides in the sample or the cyclic nucleotides conjugated with alkaline phosphatase molecules added to the incubation medium. The samples are incubated for 2 h and thereafter a substrate was added for 1 h to reveal the residual alkaline phosphatase activity in the medium, which generates a yellow product, readable on a microplate reader (Bio-rad, Laboratories Inc., Hercules, CA, USA) at 405 nm. Determination of the protein amount in the cyclic nucleotide samples was evaluated in aliquots of the neutralized acid homogenate, using the procedure of Lowry et al. [50].

### 4.8. Cellular Localization by Fluorescence Microscopy

Wild type cells grown in YPD medium to late exponential phase were transformed with GFP-tagged PDE5 constructs and selected on YPD plated supplemented with 100 μg/mL G418. Transformed cells were stained with 100 nM Mitotracker (CMTMRos MitoTracker Orange, #M7510 Thermo Fisher Scientific Inc., Waltham, MA, USA), according to manufacturer’s instructions, to visualize mitochondrial compartment. Nuclei were stained with 2.5 μg/mL DAPI (Thermo Fisher). Cells from three independent experiments were visualized using a Zeiss LSM 800 System using the 488 (EGFP) and 576 nm (MitoTracker; Thermo Fisher Scientific Inc., Waltham, MA, USA) laser lines with corresponding band-path filters. Image recording was optimized using the Zeiss Zen Software Blue edition (Carl Zeiss Microscopy GmbH, Jena, Germany). Images were acquired with a 20× PanFluor Objective (Carl Zeiss Microscopy GmbH, Jena, Germany) and imported into ImageJ (NIH, Bethesda, MD, USA), background was subtracted, and 16 fields (10 cells/field) for each condition were used to determine Pearson’s correlation coefficient (*p*) expressed as mean ± SEM. Statistical significance was calculated using the ANOVA one-way test with Holm–Sidak’s post hoc comparison. ** *p* = 0.007 (PDE5A2 vs. PDE5A1).

## 5. Conclusions

In the present work, we have thoroughly characterized murine PDE5 isoforms in *K. lactis* by analyzing their structure in vitro and their in vivo localization.

As previously reported, the three isoforms—called MmPDE5A1, MmPDE5A2, and MmPDE5A3—differ only at the N-terminus. The extra 41 amino acids of MmPDE5A1 allow it to assemble into both tetramers and dimers; the 10 extra residues of MmPDE5A2 allow it to translocate into mitochondria; MmPDE5A3, without extra residues, is dimeric only and has a mainly cytosolic expression.

MmPDE5A2’s mitochondrial localization unveiled a cAMP/cGMP signaling cascade in yeast similar to what is reported in rat mitochondria. Interestingly, an almost identical sequence is present in murine soluble adenylate cyclase type 10, known to translocate into the mitochondria [51]. Indeed, in silico analysis of MmPDE5A isoforms by MitoProt II shows the presence of a mitochondrial targeting signal in the N-terminus of MmPDE5A2. When the latter isoform is overexpressed in yeast, it induces in the host a mutation called *ragx1*, which displays the characteristics Rag^−^ phenotype of the whole complementation group (>20 genes) [28,29].

All isoforms alter cAMP/cGMP as well as NAD(P)^+^/NAD(P)H balance and affect cellular metabolism. In particular MmPDE5A2 is able to switch the metabolism from oxidative to fermentative

A closer inspection of this unexpected phenomenon highlights the role of cyclic nucleotides in the regulation of the glucose metabolism (fermentation vs. respiration) and of the energetic charge of the cell. Indeed, it is known that cAMP levels, in addition to fermentation [34], regulate the respiratory chain [42], however a role for cGMP in this same compartment was not known. By overexpressing a cGMP phosphodiesterase specifically in the mitochondria, we have altered the cAMP/cGMP ratio in control of both the mitochondrial respiratory chain and oxidative phosphorylation (Figure 5).

Given the increase in PDE5 levels observed in cardiovascular diseases [52] and in light of the growing evidence pointing to a cardioprotective effect of PDE5 inhibitors in ischemia/reperfusion injuries, mostly through the activation of the mitochondrial respiratory chain [53,54,55], the demonstration of the ability of MmPDE5A2 to influence redox status and energy balance in yeast lays the foundation for the development of targeted studies in mammalian cells.

A challenge will be the targeting of the specific mitochondrial isoform, without interfering with the PDE5 expressed in other cellular types and districts.

## Figures and Tables

**Table 1:** Primary structure of MmPDE5 N-terminus. In bold the one-letter amino acid code of the common sequence. Isoforms A1 and A3 are translated from two different start codons on the same mRNA, isoform A2 comes from an alternatively spliced exon. The result is that MmPDE5A1 has 41 amino acids more and MmPDE5A2 10 amino acids more than MmPDE5A3.

MmPDE5A1	MERAGPNSVRSQQQRDPDWVEAWLDDHRDFTFSYFIRATRD**MVNAWFSE…**
MmPDE5A2	MLPFGDKTRD**MVNAWFSE…**
MmPDE5A3	**MVNAWFSE…**
